# Angiotensin-II type 2 receptor-mediated renoprotection is independent of receptor Mas in obese Zucker rats fed high-sodium diet

**DOI:** 10.3389/fphar.2024.1409313

**Published:** 2024-07-29

**Authors:** Sanket N. Patel, Kalyani Kulkarni, Tahmid Faisal, Tahir Hussain

**Affiliations:** Department of Pharmacological and Pharmaceutical Sciences, College of Pharmacy, University of Houston, Houston, TX, United States

**Keywords:** angiotensin-II type 2 receptor, receptor Mas, high-sodium diet, C21, kidney, interdependency

## Abstract

The consumption of a high-sodium diet (HSD) is injurious and known to elevate blood pressure (BP), especially in obesity. Acute infusion studies depict a functional interdependency between angiotensin-II type 2 receptor (AT_2_R) and receptor Mas (MasR). Hence, we hypothesize that the subacute blockade of MasR should reverse AT_2_R-mediated renoprotection in obese Zucker rats (OZRs). Male OZRs were fed an HSD (for 14 days) and treated with the AT_2_R agonist C21 (100 ng/min) without or with a MasR antagonist A779 (1,000 ng/min). The indices of oxidative stress, proteinuria, kidney injury, and BP were measured before and after, along with the terminal measurements of an array of inflammatory and kidney injury markers. The HSD significantly decreased the estimated glomerular filtration rate and urinary osmolality and increased thirst, diuresis, natriuresis, kaliuresis, plasma creatinine, urinary excretion of H_2_O_2_, proteinuria, renal expression and urinary excretion of kidney injury markers (NGAL and KIM-1), and BP indexes. The HSD feeding showed early changes in the renal expression of CRP, ICAM-1, and galectin-1. The C21 treatment prevented these pathological changes. The MasR antagonist A779 attenuated C21-mediated effects on the urinary excretion and renal expression of NGAL and oxidative stress in the absence of inflammation and BP changes. Overall, we conclude that the subacute functional interactions between AT_2_R and MasR are weak or transient and that the beneficial effects of AT_2_R activation are independent of the MasR blockade in the kidney of male obese rats fed an HSD.

## 1 Introduction

The consumption of a high-sodium diet (HSD) is a major health problem not only because it is a known risk factor for salt-sensitive hypertension in obesity and kidney diseases but it can also generally offset the health benefits of an ongoing therapeutic regimen (Smyth et al., 2014). Even the consumption of an HSD for a few days can cause inflammation, oxidative stress, tubular proteinuria, and kidney injury, independent of changes in blood pressure (BP) (Teixeira et al., 2020). Furthermore, HSD consumption is a major modulator of the renin–angiotensin system (RAS). Contrary to numerous reports showing the beneficial effects of the angiotensin-II type 2 receptor (AT_2_R) and *mas* receptor (MasR) (Grobe et al., 2006; Grobe et al., 2007; Pinheiro et al., 2009; Shenoy et al., 2010), some reports show their deleterious (Ruster et al., 2011; Burrell et al., 2017; Yu et al., 2019) or lack of effect (Collister and Nahey, 2010; Dilauro et al., 2010; Jehle et al., 2012), and the interactions between AT_2_R and MasR are increasingly complex than anticipated. For instance, acute infusion studies show that the protective effects of the MasR agonist angiotensin-(1–7) (ang-(1–7)) in normotensive WKY rats and spontaneously hypertensive rats (SHRs) are dependent on AT_2_R (Walters et al., 2005; Bosnyak et al., 2012). We reported that AT_2_R and MasR are colocalized and functionally interdependent in the kidney of male obese Zucker rats (OZRs), as evident by the diuretic and natriuretic effects of the AT_2_R agonist C21 being blocked by the MasR antagonist A779, and those of the MasR agonist ang-(1–7) being blocked by the AT_2_R antagonist PD123319 (Patel et al., 2017). These cross-blocking effects of the antagonists are not due to the overlap of their affinities with these receptors. One of the mechanisms that play a role in functional interaction is the dimerization of these receptors involving a disulfide bond (Villela et al., 2015; Patel and Hussain, 2018). The functional interdependency between AT_2_R and MasR has been supported (Tesanovic et al., 2010; Kljajic et al., 2013; Hao P. et al., 2015; Hao P. P. et al., 2015; Liu et al., 2015; Papinska et al., 2015) or refuted (Santos et al., 2003; Silva et al., 2007) by various studies. However, it is unclear whether the AT_2_R-MasR interactions play a subacute/longer-term role in renal injury, inflammation, and BP regulation.

The acute (single-dose) activation/blockade of a receptor does not necessarily predict longer health outcomes. Therefore, in this study, we attempted to determine the effects of HSD intake in male genetically obese rats. Furthermore, the interactions between AT_2_R and MasR are studied, i.e., whether 14 days of treatment with the MasR antagonist A779 (1,000 ng/min) reverses the AT_2_R agonist C21 (100 ng/min)-mediated renoprotection and reduction in oxidative stress, inflammation, and BP in male HSD-fed OZRs. Male rats are generally considered vulnerable to experimental injury (Sullivan et al., 2010). Hence, it is intuitive to believe that the effect size will be pronounced in male cases and allow us to determine the deleterious effects of HSD consumption, protection upon AT_2_R stimulation, and reversal by the effects of AT_2_Rs on the blockade by the MasR antagonist. In doing so, compared to other studies that have used higher C21 concentrations, i.e., ≥1 mg/kg, we used a lower dose (0.3 mg/kg) of C21, which has been shown to exert renoprotective and anti-hypertensive effects *in vivo* (Bosnyak et al., 2010; Kemp et al., 2014). Moreover, compensatory repair processes do occur *in vivo*; hence, we compared the indexes after HSD feeding with basal values determined before commencing the treatment to infer the actual effect of HSD feeding or drug treatment.

## 2 Methods

### 2.1 Experimental protocol

Male obese Zucker rats (HsdHlr:ZUCKER-*Lepr* ^fa^, 6–7 weeks of age) weighing ∼270 g were purchased from Envigo. After arrival, the rats were housed in the University of Houston animal care facility. The animal experimental protocols used in this study were approved by the IACUC at the University of Houston and adhered to the National Institutes of Health Guide for the Care and Use of Laboratory Animals. The rats were treated without or with the AT_2_R agonist C21 (100 ng/min, Alzet 1002, 0.25 μL/h) or MasR antagonist A779 (1,000 ng/min, Alzet 2ML2, 5 μL/h) or together for 14 days using an osmotic pump (DURECT Corporation, Cupertino, CA) surgically implanted in the peritoneal cavity under isoflurane anesthesia. The dose of the AT_2_R agonist C21 was ∼0.45 mg/kg body weight and that of the MasR antagonist A779 was ∼4.17 mg/kg body weight. C21 was provided by Vicore Pharma (Sweden), and A-779 was purchased from Cayman Chemical, Ann Arbor, MI. The rats were fed a normal chow diet (ND) or HSD, (4% NaCl, Teklad TD.92034, Harlan Laboratories, Madison, WI) for 14 days. The food and water intake was monitored. Blood (∼200 µL) was collected once by pricking the tail (∼1 mm) for the preparation of plasma and baseline measurement of urea nitrogen and creatinine levels. The rats were housed in metabolic cages for 3 days before the implantation of osmotic pumps and during the terminal 3 days for urine collection and measurement of water consumption. The rats were euthanized under isoflurane anesthesia. Urine was also collected directly from the bladder, and blood was collected from a cardiac puncture for separating the plasma. The urine and plasma were stored at −80°C. The kidneys were weighed for the determination of hypertrophy and stored at −80°C for biochemical analysis.

### 2.2 General parameters

The body weight and food consumption were determined periodically. Two rats were housed per cage. Food was replenished twice a week. The rats were housed in the metabolic cages before the implantation of osmotic pumps (on days −3, −2, and −1) and after the treatment (on days 11, 12, and 13) for urine collection and measurement of water consumption. The water intake and urine output, before and after the treatment period, were presented as the mean ± standard error of the mean (SEM).

### 2.3 Inflammation array

Inflammation was assessed using sandwich-based semi-quantitative RayBio C-Series Rat Inflammation Array C3 (AAR-INF-3). In brief, kidney tissue was homogenized in a lysis buffer containing a Halt protease–phosphatase inhibitor cocktail without EDTA (78440, Thermo Fisher Scientific). The homogenate was centrifuged at 700 *g* for 1 h at 4°C. The protein concentration in the clear supernatant homogenate was measured using the Pierce™ BCA Protein Assay Kit (23225, Thermo Fisher Scientific). The membrane was incubated in a blocking buffer for 30 min, followed by incubation with a protein overnight at 4°C under gentle agitation. The sample was aspirated, and the membrane was washed with buffer 1 (5 × 5′) and buffer 2 (5 × 5′), followed by incubation with a biotinylated antibody cocktail overnight at 4°C under gentle agitation. The solution was aspirated, and the membrane was washed with both the buffers again, followed by incubation with an HRP–streptavidin antibody overnight at 4°C. After washing it again, the membrane was incubated with a chemiluminescence detection solution, and an image using the Li-Cor Odyssey Fc Imaging system was acquired. The integrated density values were normalized with two positive controls (POS1 and POS2). Normalized values of inflammatory indices are represented as the mean ± SEM.

### 2.4 Indices of kidney dysfunction

The urinary excretion of NGAL (ERLCN2) and KIM-1 (ERHAVCR1) and their expression in the kidney were determined using an ELISA kit (Thermo Fisher Scientific). The urinary excretion of NGAL and Kim-1 was presented as ng/day, and their renal expression was presented as ng/mg protein.

Urea nitrogen and creatinine levels in the urine and the plasma were measured in samples collected before the initiation of treatment and after the study period using a QuantiChrom Urea Assay Kit (DIUR-100) and EnzyChrom Creatinine Assay Kit (EIssCT-100), respectively, according to the manufacturer’s instructions (BioAssay Systems). For the urea nitrogen and creatinine measurement, 2.5 or 10 µL plasma (3kD filtrate) and urine (50×) was used, respectively. The estimated glomerular filtration rate (eGFR) was obtained based on plasma urea nitrogen and creatinine concentrations, as previously described (Besseling et al., 2021).

Urinary osmolarity and the excretion of protein, sodium, and potassium were measured in urine collected before the initiation of feeding the HSD and treatment with C21 and/or A779 (on days −3, −2, and −1) and after the study period (on days 11, 12, and 13). The urine samples were diluted 20× for osmolality and 7500× for sodium/potassium measurements. The values of 3 consecutive days were averaged and represented for the study period, i.e., “before” and “after.” Urinary osmolality was measured using an osmometer (model 3250, serial #10010827, Advanced Instruments) and presented as mOsm/kg H_2_O. The urinary excretion of sodium and potassium was estimated using an atomic absorption spectrometer (AAnalyst 400, PerkinElmer, Inc.) and presented as mmol/day (Ali et al., 2015). The excretion of protein was estimated based on the urinary protein concentration measured by the pyrogallol red-molybdate method and presented as mg/day (Patel et al., 2016).

### 2.5 Indices of oxidative stress

Urinary hydrogen peroxide levels were measured via the ADHP–peroxidase method (Patel et al., 2016). In brief, 100 µL urine (50×) was mixed with ADHP (50 μL, 20 µM) and anti-rabbit HRP IgG (50 μL, 500×) for 30 min. Fluorescence was read at an excitation wavelength of 540 nm and an emission wavelength of 590 nm using a Varioskan plate reader (Thermo Fisher Scientific) (Patel et al., 2016).

### 2.6 Tail-cuff plethysmography

BP was measured in a designated quiet area, where the rats were acclimatized for 1 h before experiments began. The BP was measured for 3 days (days −3, −2, and −1) before the surgical implantation of osmotic pumps (day 0) and during terminal 3 days (days 11, 12, and 13) at the same time between 1 p.m. and 4 p.m. by CODA 8 non-invasive tail-cuff plethysmography (Kent Scientific Corporation). The rats were acclimatized to a warm environment for 10–15 min using a warming platform (level 3). Each recording session consisted of 28 inflation and deflation cycles, of which the first three cycles were “acclimation” cycles and not used in the analysis. The minimum volume change was set as 14 µL. The average of at least five accepted runs per rat per day was used for data analysis. The daily CODA measurements acquired before the initiation of HSD feeding and treatment with C21 and/or A779 (on days −3, −2, and −1) and after the study period (on days 11, 12, and 13) were averaged separately and presented as mmHg.

### 2.7 Statistical analysis

Data are presented as the mean ± SEM. The data were analyzed using GraphPad Prism 6 and subjected to one-way or two-way ANOVA, followed by Fisher’s LSD test, whereas the values “before” and “after” were compared via a non-parametric, two-tailed, Wilcoxon matched paired *t*-test (*p* < 0.05; n = 6–7 per group). The values that fell between *p* > 0.05 and <0.1 were marked in the legend to indicate the lower level of significance in that dataset.

## 3 Results

### 3.1 General parameters

**FIGURE 1:**
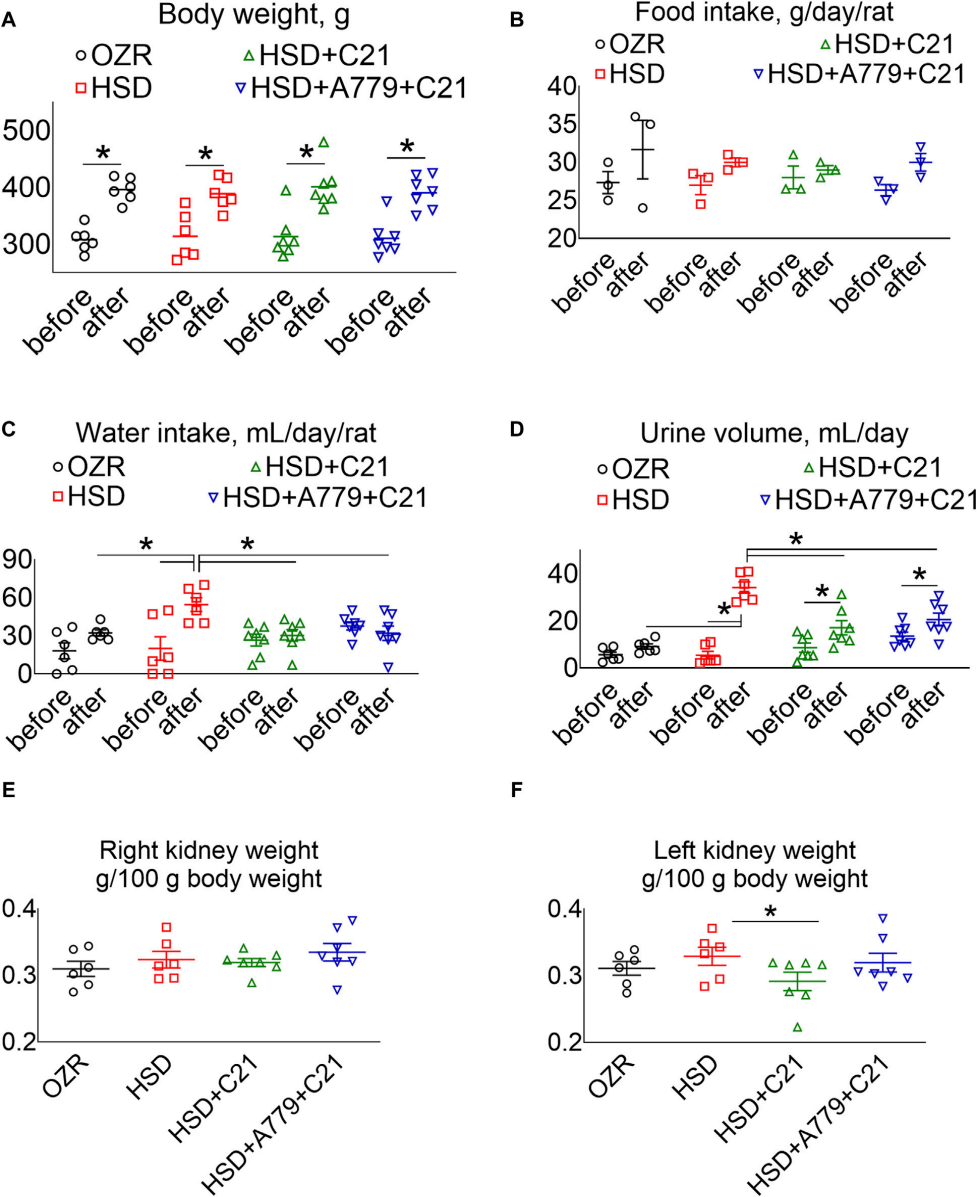
Average body weight **(A)**, food intake **(B)**, and water intake **(C)** in metabolic cages, and the urine volume **(D)** of male obese Zucker rats before the initiation of HSD feeding and treatment with the AT_2_R agonist C21 and/or *mas* receptor antagonist A779 and after the study period. The body weight normalized weight of the right **(E)** and left kidneys **(F)** of the rats are reported. The water intake was determined by housing the rats individually in a metabolic cage before (days −3, −2, and −1) and after the study period (days 11, 12, and 13). The daily food intake, water intake, and urine volume were averaged separately and represented for the study period, i.e., “before” and “after.” Food intake **(B)**: two rats were housed in a cage; hence, each value applies to two rats. The values are represented as the mean ± SEM and analyzed via two-way ANOVA, followed by Fisher’s LSD test, whereas the values “before” and “after” were compared via a non-parametric, two-tailed, Wilcoxon matched paired *t*-test; *p < 0.05, n = 6–7.

The body weight of all rats increased proportionately (Figure 1A), and the food intake remained consistent over time among all study groups (Figure 1B). As expected, the subacute consumption of the HSD significantly increased the water intake (Figure 1C) and diuresis (Figure 1D) (*p* < 0.05 before vs after and HSD vs OZRs), which was prevented by the AT_2_R agonist C21 (*p* < 0.05; HSD + C21 vs HSD). Although the consumption of the HSD did not cause renal hypertrophy *per se* (Figures 1E, F), C21 decreased the left kidney weight (*p* < 0.05, HSD + C21 vs HSD), which was reversed by the MasR antagonist A779 (*p* = ns, HSD + A779 + C21 vs C21). Otherwise, the concurrent blockade of MasR did not affect any of the above indices.

### 3.2 Indices of oxidative stress and kidney injury

**FIGURE 2:**
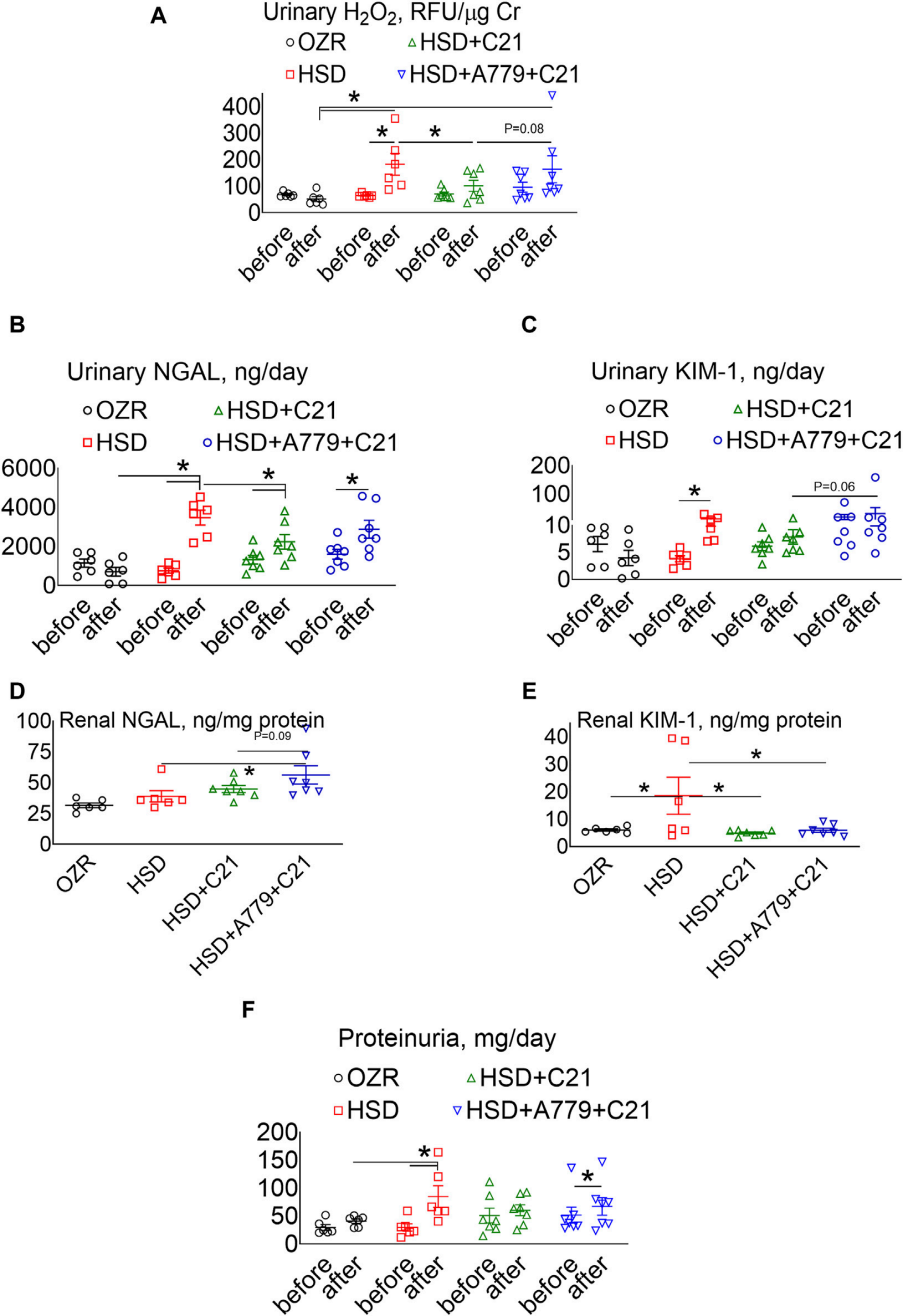
Urinary excretion of hydrogen peroxide (H_2_O_2_) **(A)**, NGAL **(B)**, and KIM-1 **(C)** and the renal expression of NGAL **(D)** and KIM-1 **(E)** and proteinuria **(F)** in male obese Zucker rats. The A–C, F indices were measured before the initiation of HSD feeding and treatment with the AT_2_R agonist C21 and/or *mas* receptor antagonist A779 and after the study period. D, E: The indices were measured terminally in the kidney homogenate after the treatment period. Proteinuria was determined in the urine collected before (days −3, −2, and −1) and after the study period (days 11, 12, and 13), averaged, and represented for the study period, i.e., “before” and “after.” The values are represented as the mean ± SEM and analyzed via two-way ANOVA **(A–C,F)** or one-way ANOVA **(D,E)**, followed by Fisher’s LSD test, whereas the values “before” and “after” were compared via a non-parametric, two-tailed, Wilcoxon matched paired *t*-test; *p < 0.05, n = 6–7.

The urinary excretion of H_2_O_2_, an indicator of oxidative stress, was increased after HSD intake (Figure 2A) (*p* < 0.05 before vs after and HSD vs OZRs). However, such an increase in the urinary H_2_O_2_ excretion was significantly prevented upon treatment with the AT_2_R agonist C21 (*p* < 0.05, HSD + C21 vs HSD, *p* = ns before vs after). The MasR blockade by A779 modestly, but not significantly, reversed the C21-mediated decrease in oxidative stress (*p =* 0.08, HSD + A779 + C21 vs HSD + C21, *p* = ns before vs after and HSD + A779 + C21 vs HSD).

The urinary excretion of NGAL (Figure 2B), a marker of renal injury, increased significantly upon HSD consumption (*p* < 0.5 before vs after and HSD vs OZRs). However, the increase in NGAL excretion was significantly less in OZRs fed the HSD treated with C21 (*p* < 0.05 before vs after and HSD + C21 vs HSD). The HSD + A779 treatment partially reversed the C21-mediated reduction in NGAL excretion (*p* < 0.05 before vs after; *p* = ns, HSD + A779 + C21 vs HSD and HSD + A779 + C21 vs HSD + C21). Similarly, the urinary excretion of KIM-1, another marker of proximal tubular brush border injury, was increased upon HSD intake (Figure 2C) (*p* < 0.05 before vs after). However, the treatment with C21 prevented such an increase in KIM-1 excretion, which was not impacted during the MasR blockade (*p* = ns both before vs after).

The renal expression of NGAL (Figure 2D) remained unchanged upon HSD intake in OZRs compared to the kidney of ND-fed OZRs and HSD + C21. However, the MasR blockade by A779 modestly increased the renal NGAL expression (*p* = 0.6 vs HSD + C21 and *p* < 0.05 vs HSD). Contrary to NGAL, the renal expression of KIM-1 (Figure 2E) was profoundly increased upon HSD intake (*p* < 0.05 HSD vs OZRs), which was prevented by treatment with C21 (*p* < 0.05 vs HSD + C21 vs HSD), but the MasR antagonist A779 did not reverse the C21-mediated reduction in renal KIM-1 expression.

Proteinuria was significantly increased after HSD intake (Figure 2F) (*p* < 0.05 before vs after and HSD vs OZRs). However, such increases in proteinuria were not evident upon treatment with the AT_2_R agonist C21 (*p* = ns before vs after, HSD + C21 vs HSD, and HSD + C21 vs OZRs). The anti-proteinuric effect of C21 was not evident when MasR was blocked with A779 (*p* < 0.05 before vs after, *p* = ns before vs after, HSD + C21 vs HSD, and HSD + C21 vs OZRs).

### 3.3 Indices of kidney dysfunction

**FIGURE 3:**
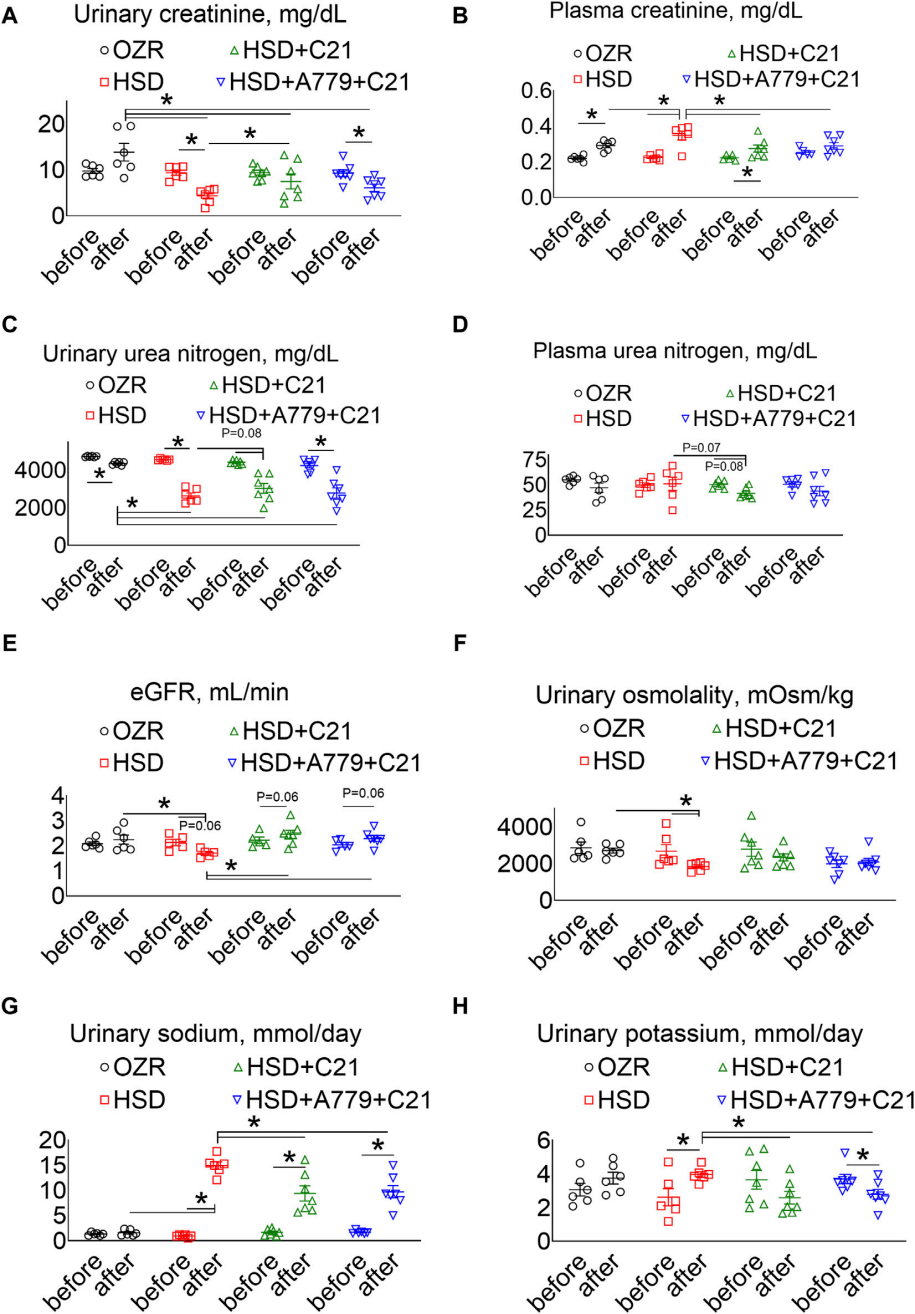
The urinary excretion of creatinine **(A)**, the plasma concentrations of creatinine **(B)**, urinary urea nitrogen **(C)**, plasma urea nitrogen **(D)**, the estimated glomerular filtration rate (eGFR) **(E)**, urinary osmolality **(F)**, urinary sodium **(G)**, and urinary potassium levels **(H)** were measured in male obese Zucker rats before the initiation of HSD feeding and treatment with the AT_2_R agonist C21 and/or *mas* receptor antagonist A779 and after the study period. The plasma values are used to calculate the eGFR, as described previously (Besseling et al., 2021). The urinary osmolality and excretion of sodium and potassium were determined in urine collected before (days −3, −2, and −1) and after the study period (days 11, 12, and 13), averaged, and represented for the study period, i.e., “before” and “after.” The values are represented as the mean ± SEM and analyzed via two-way ANOVA, followed by Fisher’s LSD test, whereas the values “before” and “after” were compared via a non-parametric, two-tailed, Wilcoxon matched paired *t*-test; *p < 0.05, n = 5–7.

As expected, the subacute consumption of the HSD caused a significant decrease in the urinary excretion of creatinine (Figure 3A), which was associated with a significant increase in the plasma creatinine levels (Figure 3B) (both *p* < 0.05 before vs after and HSD vs OZRs). The C21 treatment partially improved the urinary excretion of creatinine (*p* < 0.05 HSD + C21 vs HSD), which resulted in a decrease in the plasma creatinine levels (*p* < 0.05 before vs after and HSD + C21 vs HSD). The MasR blockade by A779 partially prevented the C21-mediated decrease in the urinary excretion of creatinine (*p* < 0.05 before vs after); however, the plasma creatinine remains unaffected (*p* < 0.5 HSD + A779 + C21 vs HSD; *p* = ns before vs after).

The urinary excretion of urea nitrogen was significantly decreased after HSD consumption (Figure 3C) (*p* < 0.05 before vs after and HSD vs OZRs), but unexpectedly, it was not associated with an increase in the plasma urea nitrogen levels (Figure 3D). However, C21 treatment partially improved urinary urea nitrogen excretion and decreased plasma urea nitrogen levels (*p* < 0.05 before vs after and *p* = 0.07 HSD vs HSD + C21). The MasR blockade by A779 partially prevented the C21-mediated decrease in the urinary excretion of urea nitrogen (*p* < 0.05 before vs after); however, the plasma urea nitrogen level remains unaffected (*p* = ns before vs after and HSD + A779 + C21 vs HSD).

The eGFR based on the plasma creatinine and urea nitrogen concentrations showed a similar effect (Besseling et al., 2021). As expected, the eGFR was significantly decreased after HSD consumption (Figure 3E) (*p* < 0.05 before vs after and HSD vs OZRs), which was modestly, but not significantly, improved with C21 treatment (*p* = 0.06 before vs after and C21 vs HSD). However, the MasR blockade did not affect the improvement in the eGFR afforded by C21.

Likewise, the HSD intake led to a predicted decrease in osmolality (Figure 3F) (*p* < 0.05 before vs after and HSD vs OZRs), which was prevented by the AT_2_R agonist C21 (*p* = ns before vs after and HSD + C21 vs HSD) but remained unaffected under the MasR blockade (*p* = ns before vs after, HSD + A779 + C21 vs HSD + C21, and HSD + A779 + C21 vs HSD).

As expected, natriuresis (Figure 3G) was increased upon HSD intake (*p* < 0.05 before vs after and HSD vs OZRs), which was decreased by C21 (*p* < 0.05 before vs after and HSD + C21 vs HSD). On the contrary, the HSD intake increased kaliuresis (Figure 3H) (*p* < 0.05 before vs after), which was significantly prevented by C21 (*p* < 0.1 HSD + C21 vs HSD; *p* = ns before vs after). The MasR antagonist A779 did not affect C21-mediated changes in natriuresis and kaliuresis (*p* < 0.05 before vs after and HSD + A779 + C21 vs HSD; *p* = ns HSD + A779 + C21 vs HSD + C21).

### 3.4 Inflammation array

**FIGURE 4:**
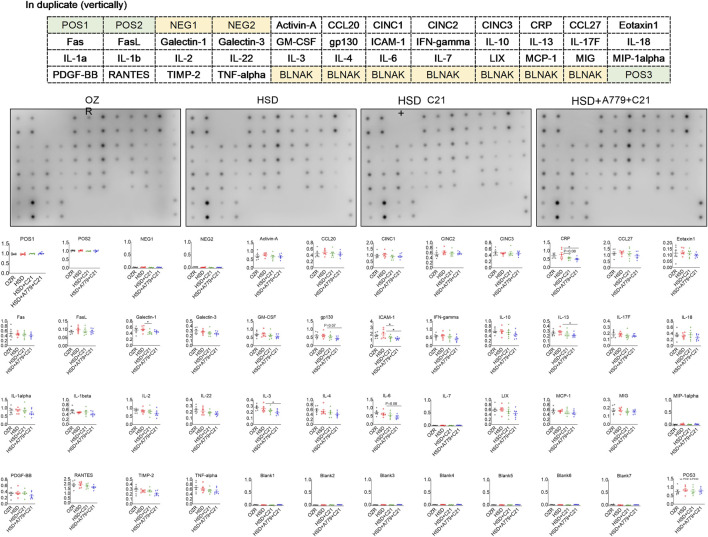
Sandwich-based semi-quantitative inflammation array in the kidney homogenate of male obese Zucker rats after the subacute feeding of HSD and treatment with the AT_2_R agonist C21 and/or MasR antagonist A779. The electrochemiluminescent integrated density values are normalized to two positive controls (POS1 and POS2). The values are represented as the mean ± SEM and analyzed via one-way ANOVA, followed by Fisher’s LSD test; *p < 0.1, n = 6.

The inflammation array detected ∼30/36 renal proteins involved in inflammation (Figure 4). HSD consumption did not statistically change these inflammatory markers compared to those found in the kidney of ND-fed OZRs. However, the treatment with the AT_2_R agonist C21 modestly reduced CRP (but not significantly with *p* = 0.08), ICAM-1 (*p* < 0.05), and galectin-1 levels (*p* < 0.05) compared with the HSD, which were not reversed during the MasR blockade by A779. Moreover, A779 further significantly reduced renal gp130, IL-13, IL-3, and IL-6 levels (*p* < 0.05 HSD + A779 + C21 vs HSD). Specifically, the renal expression of CRP, ICAM-1, and galectin-1 was associated with the renal expression of NGAL (**SI** Supplementary Table S1 and Supplementary Figure S1) and KIM-1 (Supplementary Table S2 and Supplementary Figure S2), and the renal expression of gp130, IL-13, and IL-6 was associated with changes in the plasma creatinine level and eGFR (Supplementary Table S3 and Supplementary Figure S1).

### 3.5 Blood pressure

**FIGURE 5:**
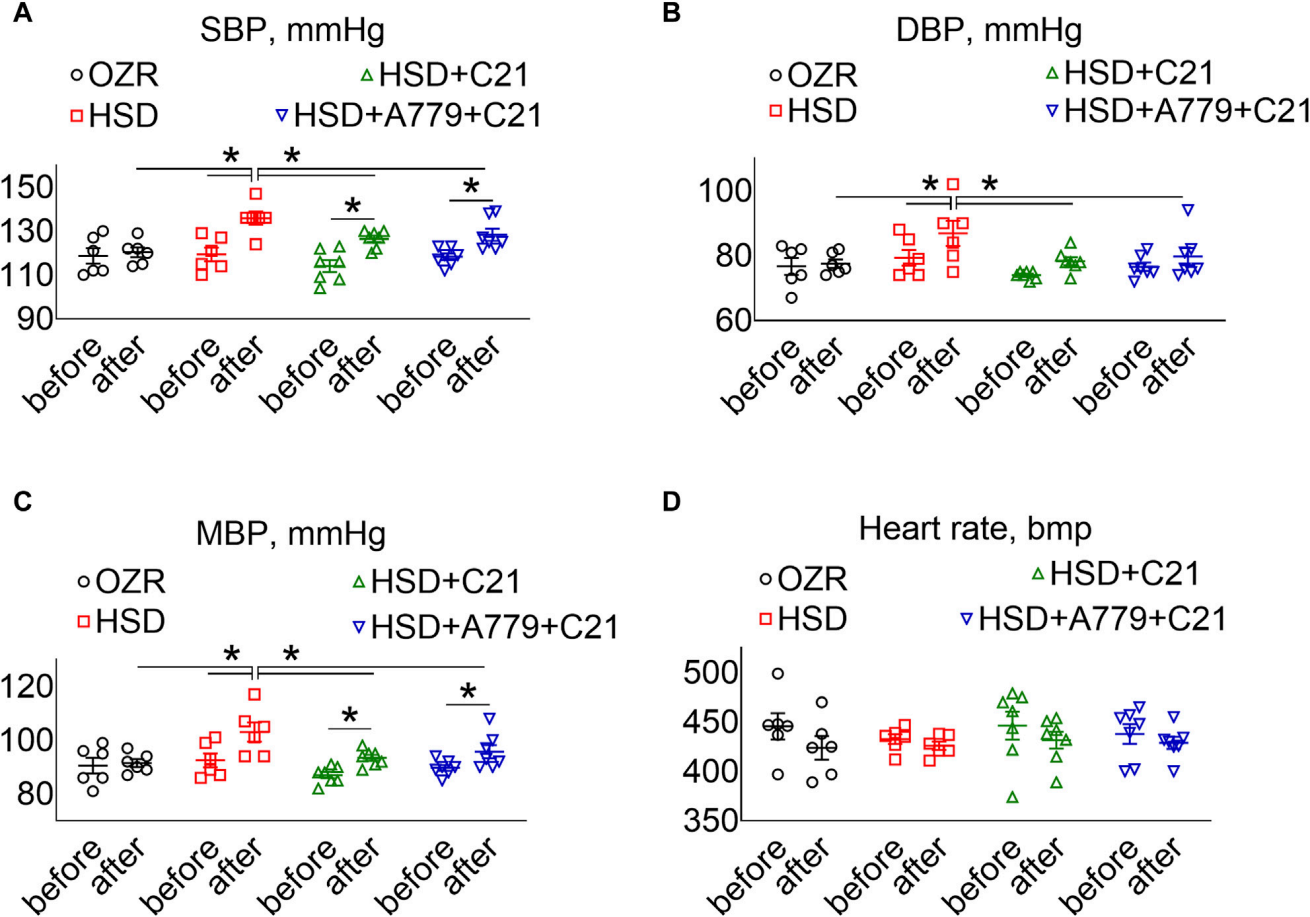
BP indexes: systolic, SBP **(A)**, diastolic, DBP **(B)**, and mean, MBP **(C)** and heart rate **(D)** measured via tail-cuff plethysmography before (days −3, −2, and −1) and after the study period (days 11, 12, and 13). The values were averaged and represented for the study period, i.e., “before” and “after.” The values are represented as the mean ± SEM and analyzed via two-way ANOVA, followed by Fisher’s LSD test, whereas the values “before” and “after” were compared via a non-parametric, two-tailed, Wilcoxon matched paired *t*-test; *p < 0.1, n = 6–7.

As expected, the systolic BP (SBP) (Figure 5A), diastolic BP (DBP) (Figure 5B), and mean BP (Figure 5C) showed a significant increase after the intake of the HSD (all *p* < 0.05 HSD vs OZRs). The C21 treatment significantly prevented the increase in these BP indexes, particularly DBP (*p* = ns before vs after) (compared to SBP, MBP, and PP; *p* < 0.05 before and after and HSD + C21 vs HSD). Similarly, the MasR antagonist A779 did not affect the C21-mediated prevention of the increase in SBP, DBP, or MBP (*p* < 0.05 before and after and HSD + A779 + C21 vs HSD). As expected, the heart rate did not change statistically among treatment groups (Figure 5D).

As a recap, diuresis–natriuresis was highly correlated with the increase in the urinary excretion of H_2_O_2_ and NGAL and a decrease in osmolality and the urinary excretion of urea nitrogen and creatinine, while the decrease in the eGFR was highly correlated with left kidney weight and plasma concentrations of urea nitrogen and creatinine (Supplementary Figure S2).

## 4 Discussion

The subacute consumption of the HSD by male OZRs increased thirst, diuresis, natriuresis, and kaliuresis; decreased the eGFR and urinary osmolality; and caused oxidative stress, inflammation, proteinuria, kidney injury and dysfunction, and hypertension. Furthermore, the treatment with the AT_2_R agonist C21 prevented these changes, which aligns with our earlier reports (Patel et al., 2016; Patel et al., 2019) but does not include MasR.

Inflammation array findings do not support the AT_2_R-MasR interdependency. The effects of AT_2_R and MasR activation on MAPK proteins involved in inflammation are dependent on the cell type they act upon and are quite opposite, suggesting the anti-inflammatory effects of AT_2_R and inflammatory effects of MasR, as our findings depict. The AT_2_R agonist C21 has been reported to exert cardioprotective effects via the phosphorylation of p38 and p42/44 MAPK (Kaschina et al., 2008). However, in rat renal proximal tubule cells, the MasR agonist ang-(1–7) reduced the phosphorylation of p38 and other MAPKs (Su et al., 2006), but in mesangial cells, ang-(1–7) induced p38 phosphorylation and fibrotic effects via MasR, which was independent of angiotensin receptors (Zimpelmann and Burns, 2009). Previously, it was reported that A779 did not affect inflammatory indexes in a rat model of pulmonary arterial hypertension (Ferreira et al., 2009). Another study utilizing a similar dose of A779 (as used here), ang-(1–7) treatment, and *masR*-null rats showed that MasR activation exerts inflammatory deleterious effects in the kidney (Esteban et al., 2009). Collectively, the findings suggest the inflammatory role of MasR in male OZR kidneys.

The MasR antagonist A779 increased the renal expression and urinary excretion of NGAL, a biomarker of kidney injury (McWilliam et al., 2014; Xiao et al., 2017), which is in contrast to the inflammation array and BP data. NGAL may appear in the urine either due to renal shedding, its reduced uptake by proximal tubules, or its enhanced glomerular clearance. Furthermore, the changes in NGAL levels could also be contributed by its release from immune cells and secretion in urine. In either case, the findings suggest that the blockade of MasR may have caused direct renal injury independent of inflammation and BP changes. The MasR antagonist A779-mediated increase in NGAL levels aligns with the inhibitory effect of the MasR agonist ang-(1–7) on NGAL (Giani et al., 2012). The renal expression of NGAL is positively associated with proteinuria and urinary excretion of H_2_O_2_ (an indicator of oxidative stress). The activation of AT_2_R (Patel et al., 2016) and MasR (Benter et al., 2008; Giani et al., 2011; Patel et al., 2016) has been shown to reduce oxidative stress in the kidney and proteinuria. Overall, it appears that the subacute functional interaction between AT_2_R and MasR is weak or transient or dependent on the pathology being studied. It is unknown whether AT_2_R and MasR can physically interact under HSD-fed conditions or upon treatment with their respective ligand. In acute settings, we reported that the MasR antagonist blocked the response of the AT_2_R agonist and *vice versa* (Patel et al., 2017). In particular, our study showed that the MasR antagonist A779 [competing with endogenous ang-(1–7)] attenuated the natriuretic/diuretic response to the AT_2_R agonist C21 in obese rats under acute settings. The AT_2_R antagonist PD123319 (competing with endogenous angII) attenuated the responses to ang-(1–7). The study showed an interdependence between AT_2_R and MasR function (Patel et al., 2017). The present study reasonably supports that AT_2_R-mediated effects were independent of MasR. However, this conclusion should be further verified as we did not include the rat groups with the MasR agonist and antagonist treatment alone. Further studies are required to understand the mechanisms of AT_2_R and MasR in the regulation of oxidative stress and proteinuria.

Moreover, the renal NGAL expression is negatively correlated with renal CRP, ICAM-1, and galectin-1 expression (Supplementary Figure S2). Hence, counterintuitively, it is likely but unclear whether A779-mediated NGAL is involved in renal recovery (Mori et al., 2005; Schroll et al., 2012) because A779 neither influenced the renal expression or urinary excretion of KIM-1, a proximal tubular scavenger receptor and another biomarker of renal tubular brush border injury (Mori et al., 2021), nor reversed AT_2_R-mediated anti-inflammation and BP indexes. Notably, renal KIM-1 is positively correlated with CRP, ICAM-1, and galectin-1 (Supplementary Figure S2). Since high salt causes low-grade inflammation, it is likely that major changes occur in the cytokine levels in this subacute setting, and the effects of C21 are not as significant as previously reported in lipopolysaccharide (LPS) and ischemia/perfusion animal models (Patel et al., 2019). It is highly likely that over time, the expression of these inflammatory markers would further increase if the consumption of the HSD continues. Contrarily, it also has been reported that NGAL and KIM-1 findings may not be correlated (Mori et al., 2021). Collectively, the findings suggest the divergent renal effects of AT_2_Rs and MasRs but not their interdependent mechanisms in renoprotection.

The decrease in HSD-induced urine osmolality could be due to the high water intake, which was reduced in the AT_2_R agonist C21-treated group, and consistently, the decrease in osmolality was prevented. Another factor that might have prevented a decrease in HSD-induced osmolality could be because AT_2_R activation inhibits various Na transporters such as Na/K-ATPase in the kidney proximal tubules (Hakam and Hussain, 2006). The effects of the Mas receptor antagonist A779 on diuresis, natriuresis, osmoregulation, eGFR, and BP regulation are acute, biphasic, transient, and often controversial (Hakam and Hussain, 2006). Subacute treatment with A779 did not completely reverse the effects of the AT_2_R agonist on the BP, diuresis, natriuresis, urinary osmolality, or eGFR, which is in slight contrast with other studies showing the renal effects of A779 in various animal models (Santos and Baracho, 1992; DelliPizzi et al., 1994; Santos et al., 1994; Handa et al., 1996; Santos et al., 1996; Simões-e-Silva et al., 1997; Simões-e-Silva et al., 1998; Widdop et al., 1999; Patel et al., 2017). Moreover, A779 was only able to transiently increase the BP in female SHRs alone (Sullivan et al., 2010). In male SHRs, A779 decreased the BP after 2 weeks of angII infusion (Sullivan et al., 2010), which aligns with our findings.

Likewise, studies utilizing the MasR agonist ang-(1–7) showed AT_2_R-independent effects in animal models of cardiovascular diseases, showing benefits, injury, or lack of protection (Dilauro et al., 2010). In a model of ischemic injury, the deletion of MasR remained beneficial in the presence of AT_2_R (Higaki et al., 2018). In an isolated aorta, it was shown that the vasodilator effects of MasR are independent of AT_2_R (Silva et al., 2007). The activation of MasR alone showed both hypertensive (Han et al., 2012; Li et al., 2015; Ren et al., 2017; Yu et al., 2019) and anti-hypertensive (Shah et al., 2012; Liang et al., 2018) effects, although the MasR agonist or antagonist was delivered to the brain (Han et al., 2012; Li et al., 2015; Ren et al., 2017; Yu et al., 2019). However, the MasR agonist ang-(1–7) itself did show protective effects in DOCA-salt (Grobe et al., 2006) or angII-induced (Grobe et al., 2007) hypertensive rats but were independent of the changes in BP. We showed an interaction of AT_2_R and MasR in the proximal tubules in the kidney and *in vitro*. We speculate that such an interaction exists in other cell types as well as these receptors are expressed on the endothelial, epithelial, and vascular smooth muscle cells and share similar signaling and functions such as vasodilatation and natriuresis (Patel and Hussain, 2018). Salt is known to cause damage to the endothelial cells, so the function of these receptors on these cells would likely be impacted, contributing to the blood pressure through the impact on the vascular tone. However, due to the anti-oxidative and anti-inflammatory functions of AT_2_R, it is likely that endothelial function is protected. However, in the long-term study, this interaction, if any, does not appear to alter the AT_2_R-mediated renoprotection, but studies to evaluate the physical interaction in various cell types are needed to determine their functional dependence or independence.

Based on the affinity of angiotensin peptides (Bosnyak et al., 2011) and considering the notion that the dose of an antagonist should be ∼10-fold higher than the Ki of the receptor to identify the measurable *in vivo* response (Macari et al., 1993; Walters et al., 2005), the doses of C21 and A779 we used are within the range of their specificity. Moreover, A779 is a selective MasR antagonist and does not interact with angiotensin receptors; hence, A779 is a better candidate to use to determine interdependency between AT_2_R and MasR. Likewise, the lower doses of A779 neither affected BP (Walters et al., 2005; Grobe et al., 2007; Collister and Nahey, 2010) nor were consistent in showing the effects of MasR on diuresis–natriuresis (Santos and Baracho, 1992; Santos et al., 1994; Simões-e-Silva et al., 1997; Simões-e-Silva et al., 1998; Widdop et al., 1999). Hence, we can conclude that the lack of reversibility by A779 on BP, diuresis, natriuresis, urinary osmolality, or eGFR is not due to the dose of A779 used in this study.

Earlier, Walters et al. (2005) showed that in AT_1_R antagonist-treated rats, ang-(1–7) mediated vasodepressor effects through the activation of AT_2_R and formation of nitric oxide. Later, it was found that ang-(1–7)/MasR is an AT_1_R antagonist (Mahon et al., 1994; Kostenis et al., 2005), but ang-(1–7) is also a biased AT_1_R agonist via beta-arrestin (Galandrin et al., 2016; Teixeira et al., 2017). We observed that ang-(1–7) synergizes the nitric oxide response of AT_2_R agonists in human renal proximal tubule epithelial (human kidney-2) cells, which is dependent on AT_1_R and AT_2_R but not MasR (Patel and Hussain, 2020). However, contrary to these findings, previous reports show that A779 did not reverse AT_2_R agonist (Ocaranza et al., 2014) or AT_1_R antagonist (Varagic et al., 2013)-mediated anti-hypertensive effects. Further studies are required to dissect the long-term *in vivo* interactions among AT_1_R, AT_2_R, and MasR.

The extensive literature does show the direct causal relationship between HSD intake and the worsening of hypertensive proteinuric kidney injury independent of comorbidities. However, the degree of injury may differ among individuals. Moreover, globally, the consumption of the HSD has increased over time, and the consumption of an HSD over time can offset the health benefits of ongoing therapeutic regimens. Therefore, considering the therapeutic implications of an ∼10-fold lower dose of AT_2_R agonist C21 (compared to the typically used dose of C21) in renoprotection in male obese rats, the current work holds high significance. It is important to note that despite numerous reports describing the beneficial role of the MasR agonist ang-(1–7), we show that the MasR blockade did not alter the BP indexes and renal inflammation, and at large, the subacute effects of the AT_2_R agonist C21 are independent of MasR. The AT_2_R-MasR functional interdependence appears transient, and MasR may neither be a potent regulator of BP nor has a sustained effect on BP, which is a dynamic phenomenon regulated by multiple organ systems. In addition, the findings show that MasR may play a direct role in the regulation of NGAL and oxidative stress independent of inflammation and BP regulation. Although the present study suggests that AT_2_R-mediated beneficial effects are independent of the functional interaction with MasR in the subacute setting, other components of the RAS, particularly a reduction in angII/AT_1_R and increase in ang-(1–7) in the kidney, may play a role in the net effects of AT_2_R activation, as reported earlier (Samuel et al., 2012; Patel et al., 2017). Although we did not measure RAS components, particularly angII and AT_1_R expression in the kidney of the C21 + A779 group, it is likely that the C21 effects on angII/AT_1_R reduction will continue even in the presence of A779, which does not alter the C21 effects in the present study.

## 5 New and noteworthy

The subacute consumption of the HSD caused renal injury, as evident by KIM-1 and NGAL levels. KIM-1 has shown a positive correlation with CRP, ICAM-1, and galectin-1, while NGAL has shown a negative correlation with CRP, ICAM-1, and galectin-1. The AT_2_R agonist C21 prevented the subacute deleterious effects of HSD consumption in male OZRs, which are not reversed by the MasR antagonist A779. However, A779 increased proteinuria, oxidative stress, and NGAL, independent of inflammation, BP changes, and AT_2_R.

## Data Availability

The original contributions presented in the study are included in the article/Supplementary Material; further inquiries can be directed to the corresponding author.
