# Supplementary material for: Angiotensin-II type 2 receptor-mediated renoprotection is independent of receptor Mas in obese Zucker rats fed high-sodium diet
Source: Front Pharmacol. 2024 Jul 29;15:1409313. doi: 10.3389/fphar.2024.1409313 (PMC11317439; doi:10.3389/fphar.2024.1409313)
Supplement: Supplementary file 1 [file DataSheet1.docx]

**SI Appendix**

**Angiotensin-II type 2 receptor-mediated renoprotection is independent of receptor Mas in obese Zucker rats fed high sodium diet**

**Materials and Methods**

***General parameters***

The body weight and food consumption was determined periodically. Two rats were housed per cage. Food was replenished twice a week. Rats were housed in the metabolic cages before the implantation of osmotic pumps (on days -3, -2, and -1) and after the treatment (on days 11, 12, and 13) for urine collection and measurement of water consumption. The water intake and urine output, before and after, the treatment period was normalized and presented as mean ± standard error of the mean (SEM).

***Inflammation array***

Inflammation was assessed using sandwich-based semi-quantitative RayBio C-Series Rat Inflammation Array C3 (AAR-INF-3). Briefly, kidney tissue was homogenized in a lysis buffer containing Halt protease-phosphatase inhibitor cocktail without EDTA (78440, ThermoFisher Scientific). Homogenate was centrifuged at 700g for 1 hour at 4°C. Protein concentration was measured in clear supernatant homogenate using Pierce™ BCA Protein Assay Kit (23225, ThermoFisher Scientific). The membrane was blocked in a blocking buffer for 30 minutes. Protein was incubated overnight at 4°C with gentle agitation. The sample was aspirated and the membrane was washed with buffer 1 (5X5’) and buffer 2 (5X5’) followed by incubation with a biotinylated antibody cocktail overnight at 4°C with gentle agitation. The solution was aspirated and the membrane was washed with buffer 1 (5X5’) and buffer 2 (5X5’) followed by incubation with HRP-streptavidin antibody overnight at 4°C. The solution was aspirated and the membrane was washed with buffer 1 (5X5’) and buffer 2 (5X5’). The membrane was incubated with a chemiluminescence detection solution (1:1 mixture of detection buffer C and D to acquire an image using the Li-Cor Odyssey Fc Imaging system. The integrated density values were normalized with two positive controls (POS1 and POS2) and normalized values of inflammatory indices are represented as mean ± SEM.

***Indices of kidney dysfunction***

The urinary excretion of NGAL (ERLCN2) and KIM-1 (ERHAVCR1) and their expression in the kidney were determined by an ELISA kit (Thermo Fisher Scientific). The urinary excretion of NGAL and Kim-1 was presented as ng/day and their renal expression was presented as ng/mg protein.

Urea nitrogen and creatinine in the urine and the plasma were measured in samples collected before the initiation of treatment and after the study period via QuantiChrom urea assay kit (DIUR-100) and EnzyChrom Creatinine assay kit (EIssCT-100), respectively, according to manufacturer’s instructions (BioAssay Systems). For the urea nitrogen and creatinine measurement, 2.5 µL or 10 µL plasma (3kD filtrate) or urine (50X) was used, respectively. The glomerular filtration rate was estimated (eGFR) based on plasma urea nitrogen and creatinine concentrations as previously described.[^1^](#_ENREF_1)

Urinary osmolarity, the excretion of protein, sodium, and potassium was measured in urine collected before the initiation of feeding HSD and treatment with C21 and/or A779 (on days -3, -2, and -1), and, after the study period (on days 11, 12, and 13). Urine samples were diluted 20X for osmolality and 7500X for sodium/potassium measurements, respectively. The daily values were averaged and represented for the study period, i.e., “before” and “after”. Urinary osmolality was measured using the Osmometer (model 3250, serial #10010827, Advanced Instruments) and presented as mOsm/kg H_2_O. The excretion of sodium and potassium was estimated using an atomic absorption spectrometer (AAnalyst 400, PerkinElmer, Inc.) and presented as mg/day.[^2^](#_ENREF_2) The excretion of protein was estimated based on urinary protein concentration measured via the pyrogallol red-molybdate method and presented as mg/day.[^3^](#_ENREF_3)

***Indices of oxidative stress***

Urinary hydrogen peroxide was measured via the ADHP-peroxidase method.[^3^](#_ENREF_3) Briefly, 100 µL urine (50X) was mixed with ADHP (50 µL, 20 µM) and anti-rabbit HRP IgG (50 µL, 500X) for 30 minutes. Fluorescence was read at an excitation wavelength of 540 nm and emission wavelength of 590 nm using a Varioskan plate reader (Thermo Fisher Scientific).[^3^](#_ENREF_3)

***Tail-cuff plethysmography***

The blood pressure (BP) was measured in a designated quiet area, where mice acclimatized for 1 hour before experiments began. The BP was measured for 3 days (-3, -2, and -1 day) before the surgical implantation of osmotic pumps (day 0) and during terminal 3 days (d11, d12, and d13) at the same time between 1 pm to 4 pm using CODA 8 non-invasive tail-cuff plethysmography (Kent Scientific Corporation). The rats were acclimatized to a warm environment for 10-15 minutes using a warming platform (level 3). Each recording session consisted of 28 inflation and deflation cycles, of which the first 3 cycles were “acclimation” cycles and were not used in the analysis. The minimum volume change was set as 14 µL. The average of at least 5 accepted run per rat per day was used for data analysis. The daily CODA measurements acquired before the initiation of HSD feeding and treatment with C21 and/or A779 (on days -3, -2, and -1), and after the study period (on days 11, 12, and 13) were averaged and presented as mmHg.

| Table 1. Correlation of renal NGAL (ng/mg protein) with an expression of cytokines in the kidney of male obese Zucker rats. | | | |
| --- | --- | --- | --- |
| Renal NGAL (ng/mg protein). | Pearson r | R squared | p-value (two-tailed) |
| Activin-A | -0.05149 | 0.003 | 0.8112 |
| CCL20 | -0.0504 | 0.003 | 0.8151 |
| CINC1 | -0.09121 | 0.008 | 0.6716 |
| CINC2 | -0.03064 | 0.001 | 0.887 |
| CINC3 | -0.2301 | 0.053 | 0.2795 |
| CRP | -0.4396 | **0.193** | **0.0316** |
| CCL27 | -0.0267 | 0.001 | 0.9014 |
| Eotaxin1 | -0.2199 | 0.048 | 0.3018 |
| Fas | -0.0726 | 0.005 | 0.736 |
| FasL | -0.1323 | 0.018 | 0.5376 |
| Galectin-1 | -0.488 | **0.238** | **0.0156** |
| Galectin-3 | -0.2934 | 0.086 | 0.1641 |
| GM-CSF | -0.2576 | 0.066 | 0.2243 |
| gp130 | -0.2084 | 0.043 | 0.3284 |
| ICAM-1 | -0.5 | **0.250** | **0.0129** |
| IFN-gamma | -0.07426 | 0.006 | 0.7302 |
| Il-10 | -0.07355 | 0.005 | 0.7327 |
| Il-13 | -0.1361 | 0.019 | 0.5259 |
| Il-17F | -0.2605 | 0.068 | 0.2189 |
| Il-18 | 0.09923 | 0.010 | 0.6446 |
| Il-1a | -0.1805 | 0.033 | 0.3986 |
| Il-1b | -0.2486 | 0.062 | 0.2415 |
| Il-2 | -0.2453 | 0.060 | 0.248 |
| Il-22 | -0.5312 | **0.282** | **0.0076** |
| Il-3 | -0.5128 | **0.263** | **0.0104** |
| Il-4 | -0.2613 | 0.068 | 0.2174 |
| Il-6 | -0.1911 | 0.037 | 0.3709 |
| LIX | -0.01738 | 0.000 | 0.9357 |
| MCP-1 | -0.09194 | 0.008 | 0.6692 |
| MIG | -0.01739 | 0.000 | 0.9357 |
| PDGF-BB | -0.05285 | 0.003 | 0.8062 |
| RANTES | -0.304 | 0.092 | 0.1487 |
| TIMP-2 | -0.2725 | 0.074 | 0.1977 |
| TNF-alpha | -0.3159 | 0.100 | 0.1326 |

**
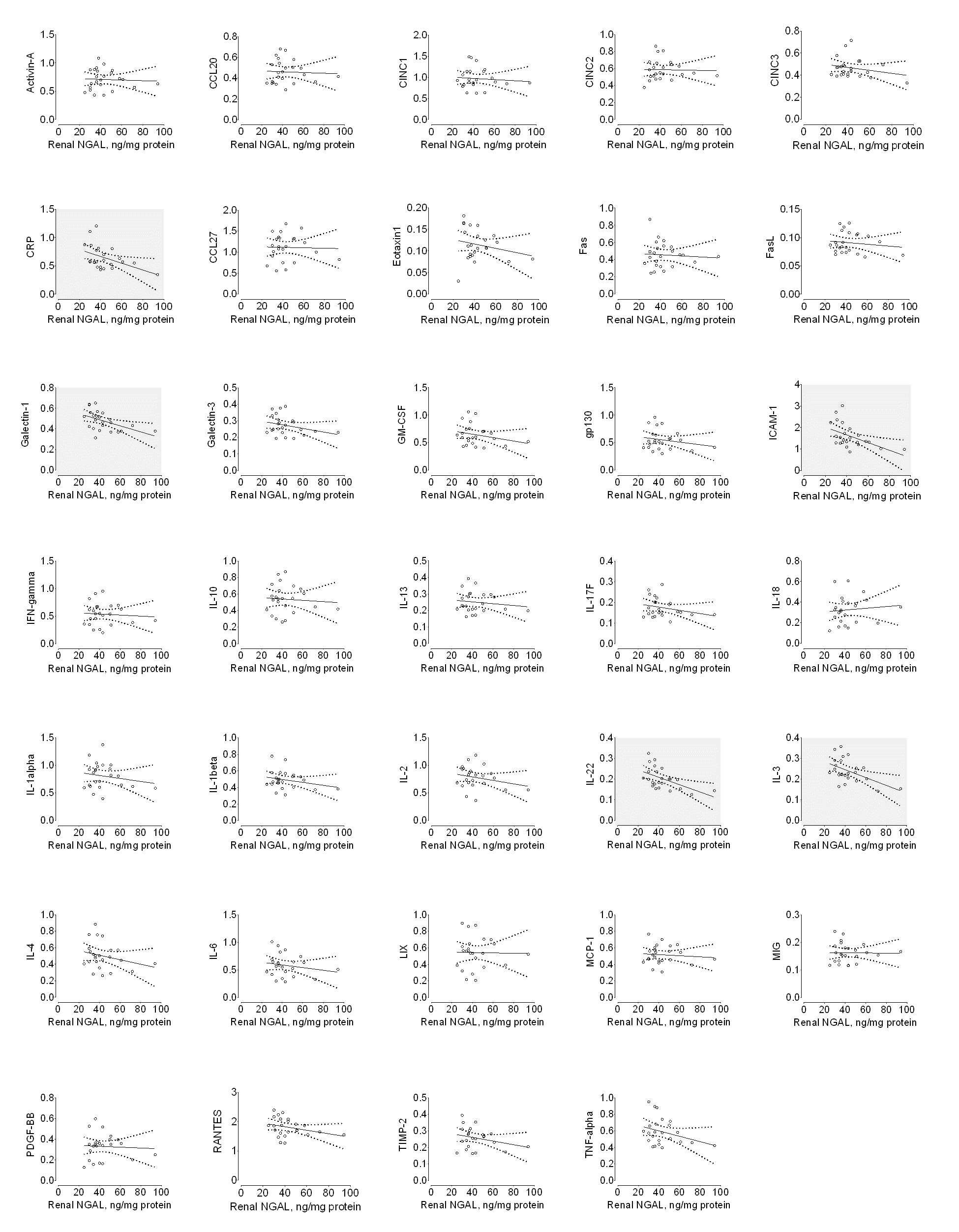
**

| Table 2. Correlation of renal KIM-1 (ng/mg protein) with an expression of cytokines in the kidney of male obese Zucker rats. | | | |
| --- | --- | --- | --- |
| Renal KIM-1 (ng/mg protein). | Pearson r | R squared | p-value (two-tailed) |
| Activin-A | 0.07923 | 0.006 | 0.7129 |
| CCL20 | 0.01716 | 0.000 | 0.9366 |
| CINC1 | 0.2055 | 0.042 | 0.3353 |
| CINC2 | 0.3387 | 0.115 | 0.1054 |
| CINC3 | 0.05225 | 0.003 | 0.8084 |
| CRP | 0.5657 | **0.320** | **0.004** |
| CCL27 | -0.004347 | 0.000 | 0.9839 |
| Eotaxin1 | 0.1699 | 0.029 | 0.4275 |
| Fas | 0.07312 | 0.005 | 0.7342 |
| FasL | 0.04877 | 0.002 | 0.821 |
| Galectin-1 | 0.3989 | **0.159** | **0.0535** |
| Galectin-3 | 0.01594 | 0.000 | 0.9411 |
| GM-CSF | 0.2499 | 0.062 | 0.239 |
| gp130 | 0.212 | 0.045 | 0.32 |
| ICAM-1 | 0.6274 | **0.394** | **0.001** |
| IFN-gamma | 0.08969 | 0.008 | 0.6768 |
| Il-10 | 0.05273 | 0.003 | 0.8067 |
| Il-13 | 0.2983 | 0.089 | 0.1568 |
| Il-17F | 0.1714 | 0.029 | 0.4233 |
| Il-18 | -0.2162 | 0.047 | 0.3103 |
| Il-1a | -0.01854 | 0.000 | 0.9315 |
| Il-1b | -0.1055 | 0.011 | 0.6236 |
| Il-2 | 0.1451 | 0.021 | 0.4986 |
| Il-22 | 0.3763 | **0.142** | **0.0699** |
| Il-3 | 0.3766 | **0.142** | **0.0697** |
| Il-4 | 0.2823 | 0.080 | 0.1814 |
| Il-6 | 0.1406 | 0.020 | 0.5123 |
| LIX | 0.05986 | 0.004 | 0.7811 |
| MCP-1 | 0.0728 | 0.005 | 0.7353 |
| MIG | 0.3704 | **0.137** | **0.0748** |
| PDGF-BB | 0.1555 | 0.024 | 0.4682 |
| RANTES | 0.2682 | 0.072 | 0.2051 |
| TIMP-2 | 0.03344 | 0.001 | 0.8767 |
| TNF-alpha | 0.2872 | 0.082 | 0.1736 |

**
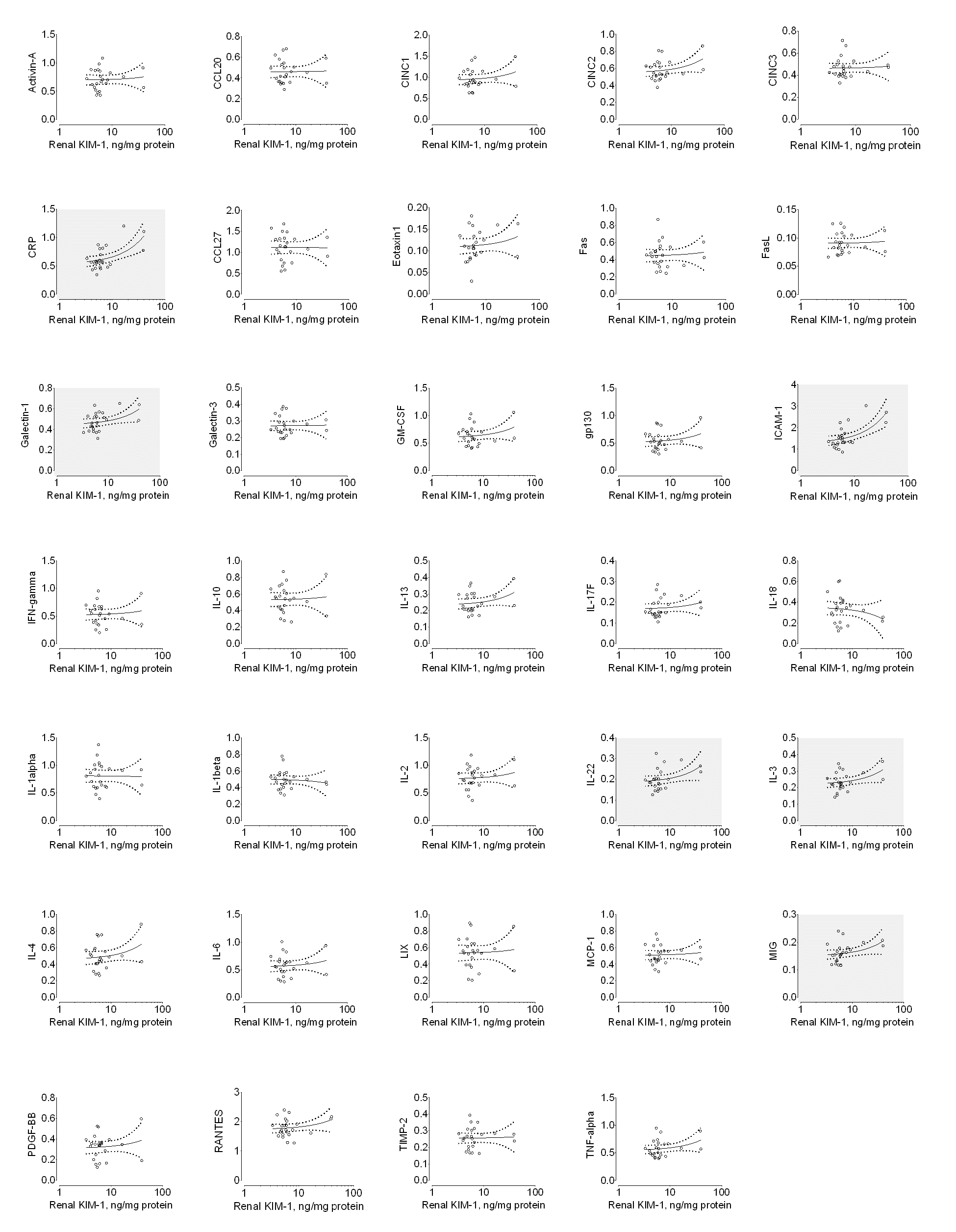
**

| Table 3. Correlation of estimated glomerular filtration rate (mL/min/g kidney) with an expression of cytokines in the kidney of male obese Zucker rats. | | | |
| --- | --- | --- | --- |
| eGFR (ml/min/g kidney) vs. | Pearson r | R squared | p-value (two-tailed) |
| Activin-A | -0.6562 | **0.431** | **0.0005** |
| CCL20 | -0.5975 | **0.357** | **0.002** |
| CINC1 | -0.6008 | **0.361** | **0.0019** |
| CINC2 | -0.4423 | **0.196** | **0.0305** |
| CINC3 | -0.3062 | 0.094 | 0.1456 |
| CRP | 0.1374 | 0.019 | 0.522 |
| CCL27 | -0.4751 | **0.226** | **0.019** |
| Eotaxin1 | -0.0255 | 0.001 | 0.9058 |
| Fas | -0.4979 | **0.248** | **0.0133** |
| FasL | -0.6426 | **0.413** | **0.0007** |
| Galectin-1 | -0.375 | 0.141 | 0.071 |
| Galectin-3 | -0.4334 | **0.188** | **0.0344** |
| GM-CSF | -0.4355 | **0.190** | **0.0334** |
| gp130 | -0.4987 | **0.249** | **0.0131** |
| ICAM-1 | 0.01752 | 0.000 | 0.9353 |
| IFN-gamma | -0.4625 | **0.214** | **0.0229** |
| Il-10 | -0.4812 | **0.232** | **0.0173** |
| Il-13 | -0.4387 | **0.192** | **0.032** |
| Il-17F | -0.4146 | **0.172** | **0.044** |
| Il-18 | -0.4104 | **0.168** | **0.0464** |
| Il-1a | -0.4377 | **0.192** | **0.0324** |
| Il-1b | -0.4004 | **0.160** | **0.0525** |
| Il-2 | -0.4686 | **0.220** | **0.0209** |
| Il-22 | -0.2401 | 0.058 | 0.2583 |
| Il-3 | -0.2312 | 0.053 | 0.277 |
| Il-4 | -0.4055 | **0.164** | **0.0493** |
| Il-6 | -0.4099 | **0.168** | **0.0467** |
| LIX | -0.4871 | **0.237** | **0.0158** |
| MCP-1 | -0.4936 | **0.244** | **0.0142** |
| MIG | -0.2524 | 0.064 | 0.234 |
| PDGF-BB | -0.385 | **0.148** | **0.0632** |
| RANTES | -0.4012 | **0.161** | **0.052** |
| TIMP-2 | -0.3858 | **0.149** | **0.0626** |
| TNF-alpha | -0.5418 | **0.294** | **0.0062** |

**
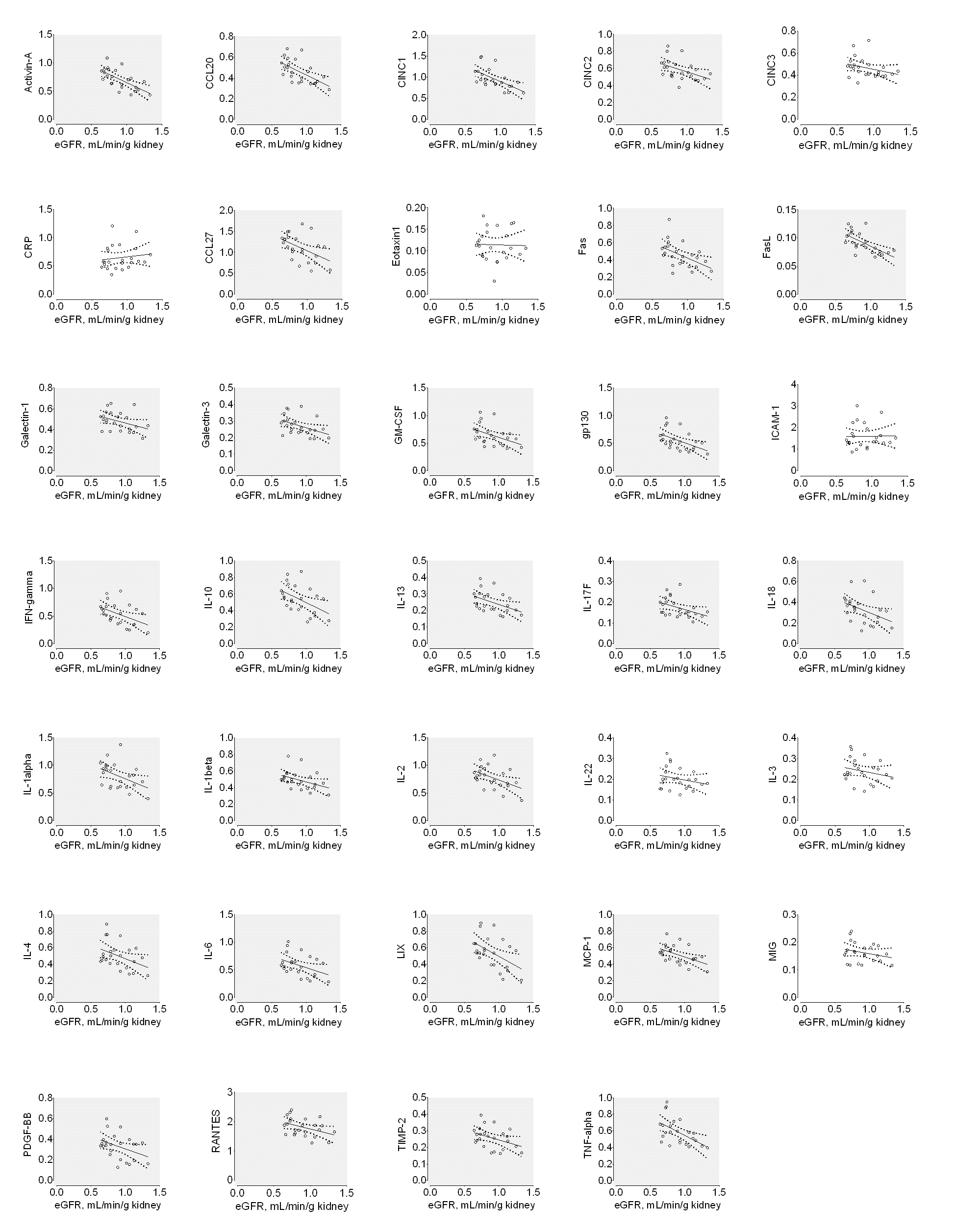
**

**
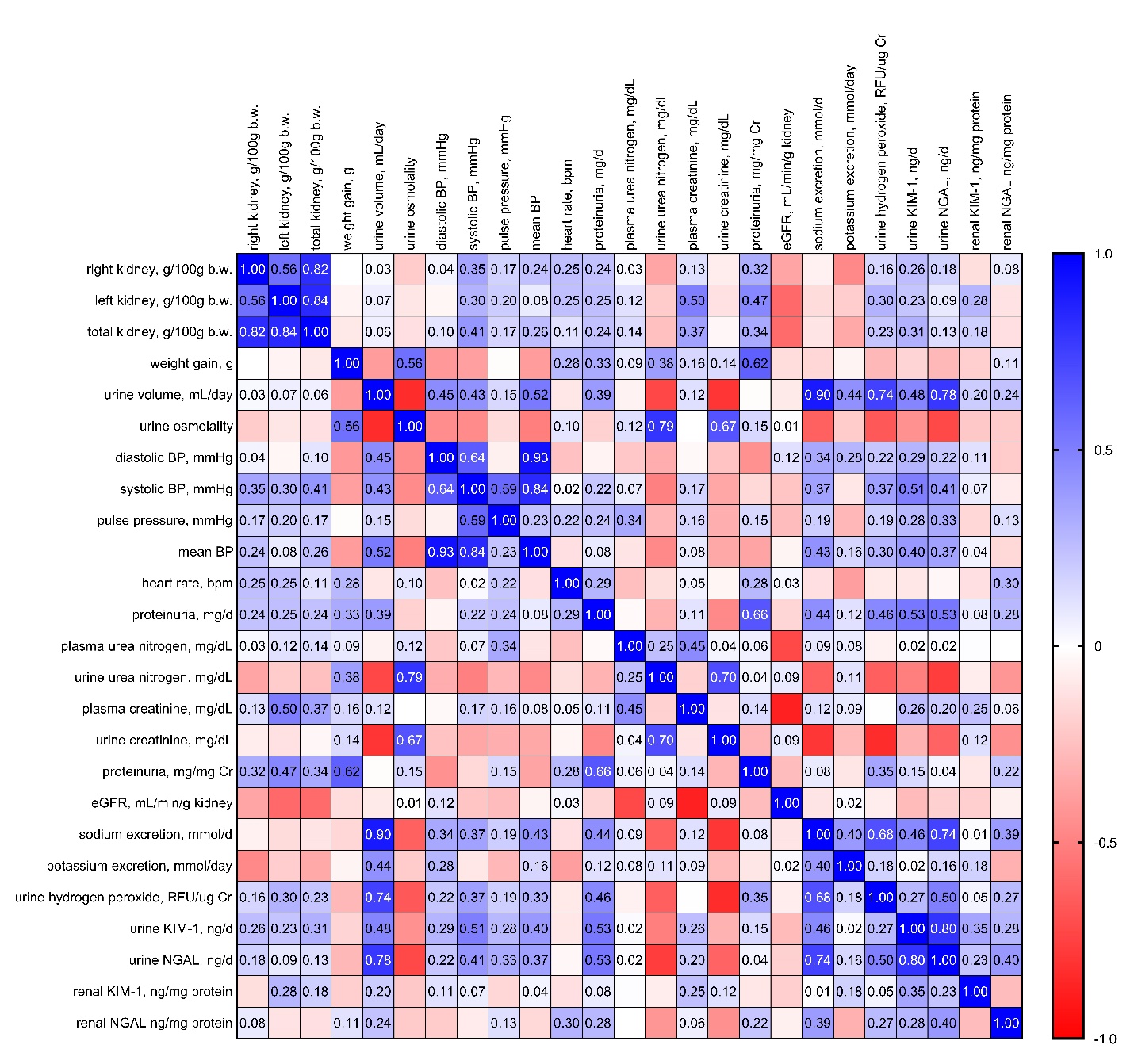
**

**SI Figure 1.**

**
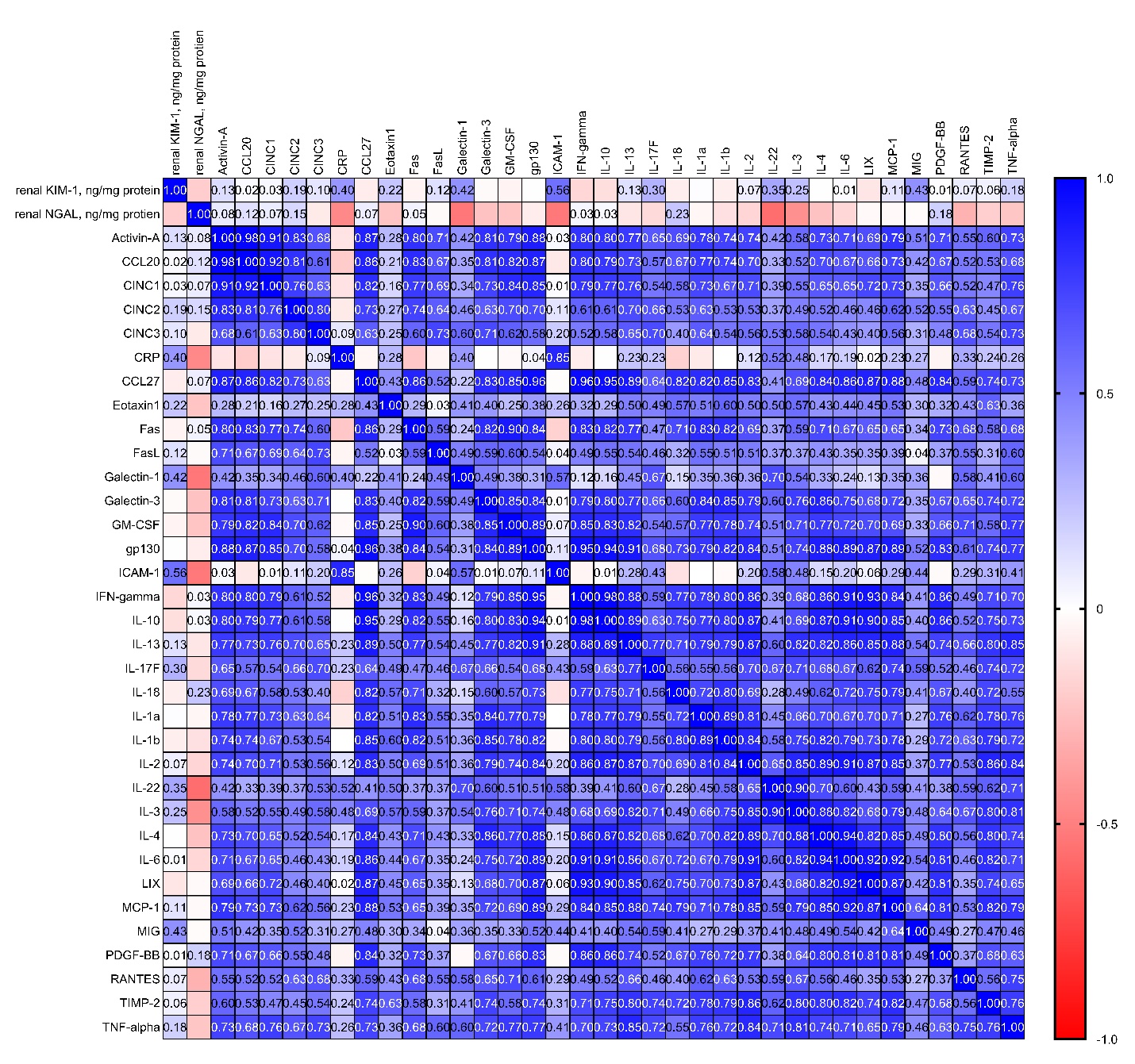
SI Figure 2.**

**REFERENCES**

[1] Besseling PJ, Pieters TT, Nguyen ITN*, et al.* A plasma creatinine- and urea-based equation to estimate glomerular filtration rate in rats. *Am J Physiol Renal Physiol* 2021;320:F518-F24.

[2] Ali Q, Patel S, Hussain T. Angiotensin AT2 receptor agonist prevents salt-sensitive hypertension in obese Zucker rats. *Am J Physiol Renal Physiol* 2015;308:F1379-85.

[3] Patel SN, Ali Q, Hussain T. Angiotensin II Type 2-Receptor Agonist C21 Reduces Proteinuria and Oxidative Stress in Kidney of High-Salt-Fed Obese Zucker Rats. *Hypertension* 2016;67:906-15.

[4] Rieg T. A High-throughput method for measurement of glomerular filtration rate in conscious mice. *J Vis Exp* 2013:e50330.
